# Experimental cross-contamination of chicken salad with *Salmonella enterica* serovars Typhimurium and London during food preparation in Cambodian households

**DOI:** 10.1371/journal.pone.0270425

**Published:** 2022-08-01

**Authors:** Rortana Chea, Hung Nguyen-Viet, Sothyra Tum, Fred Unger, Johanna Lindahl, Delia Grace, Chhay Ty, Sok Koam, Vor Sina, Huy Sokchea, Son Pov, Theng Heng, Or Phirum, Sinh Dang-Xuan

**Affiliations:** 1 National Animal Health and Production Research Institute, General Directorate of Animal Health and Production, Phnom Penh, Cambodia; 2 International Livestock Research Institute, Nairobi, Kenya; 3 Department of Biomedical Sciences and Veterinary Public Health, Swedish University of Agricultural Sciences, Uppsala, Sweden; 4 Department of Clinical Sciences, Swedish University of Agricultural Sciences, Uppsala, Sweden; 5 Department of Medical Biochemistry and Microbiology, Uppsala University, Uppsala, Sweden; 6 Natural Resources Institute, University of Greenwich, Kent, United Kingdom; 7 Livestock Development for Community Livelihood Organization, Phnom Penh, Cambodia; 8 Center for Public Health and Ecosystem Research, Hanoi University of Public Health, Hanoi, Vietnam; Guru Angad Dev Veterinary and Animal Sciences University, INDIA

## Abstract

Non-typhoidal *Salmonellae* are common foodborne pathogens that can cause gastroenteritis and other illnesses in people. This is the first study to assess the transfer of *Salmonella enterica* from raw chicken carcasses to ready-to-eat chicken salad in Cambodia. Twelve focus group discussions in four Cambodian provinces collected information on typical household ways of preparing salad. The results informed four laboratory experiments that mimicked household practices, using chicken carcasses inoculated with *Salmonella*. We developed four scenarios encompassing the range of practices, varying by order of washing (chicken or vegetables first) and change of chopping utensils (same utensils or different). Even though raw carcasses were washed twice, *Salmonella* was isolated from 32 out of 36 chicken samples (88.9%, 95% CI: 73.0–96.4) and two out of 18 vegetable samples (11.1%, 95% CI: 1.9–36.1). *Salmonella* was detected on cutting boards (66.7%), knives (50.0%) and hands (22.2%) after one wash; cross-contamination was significantly higher on cutting boards than on knives or hands (*p*-value < 0.05). The ready-to-eat chicken salad was contaminated in scenario 1 (wash vegetables first, use same utensils), 2 (wash vegetables first, use different utensils) and 3 (wash chicken first, use same utensils) but not 4 (wash chicken first, use different utensils) (77.8%, 11.1%, 22.2% and 0%, respectively). There was significantly higher *Salmonella* cross-contamination in scenario 1 (wash vegetables first, use same utensils) than in the other three scenarios. These results show how different hygiene practices influence the risk of pathogens contaminating chicken salad. This information could decrease the risk of foodborne disease in Cambodia and provides inputs to a quantitative risk assessment model.

## Introduction

Food safety is a major concern worldwide [1]. Animal-source food (ASF) provides essential nutrients but is a common source of pathogens. The World Health Organization estimated that, in 2010, more than 600 million illnesses were caused by 31 common foodborne hazards: most were diarrhoeal and caused by zoonoses [2]. Foodborne disease (FBD) affects humans of all age groups, but children under five years are among the most vulnerable. According to the Foodborne Disease Burden Epidemiology Reference Group (FERG), one in 10 children worldwide suffer from FBD annually [3]. FBD also decreases human capital, entails prevention and treatment costs, and hinders trade [4]; economic losses are estimated at more than USD 100 billion a year across developing countries [1]. In Cambodia, over 5,000 people fell sick from FBD in 371 outbreaks in 2019 [5], but this is a huge underestimate as there is inadequate surveillance of FBD [6, 7].

*Salmonella* spp. is one of the most important causes of FBD and is often associated with ASF consumption [8]. Non-typhoidal *Salmonella* (NTS) was estimated to cause 59,000 deaths out of 420,000 annual deaths globally from foodborne hazards in 2010 [3].

Chicken is popular in Cambodia; it is affordable, widely available and provides protein and micronutrients essential for growth and health [9, 10]. In 2020, total meat consumption was 301,000 tons per year, of which poultry meat was 62,000 tons [11]. Poultry meat consumption is expected to increase by 5.5% annually due to increased demand driven by population growth, urbanization and increasing incomes [12]. Cambodians prefer local, backyard chicken, but the modern industrial sector, which uses exotic chicken, has higher productivity and is growing rapidly [13]. High and rising consumption of chicken meat is a health concern because it is a common source of *Salmonella* and other pathogens [14, 15]. *Salmonella* can persist in the chicken intestinal tract without causing clinical signs and is not detected by meat inspection. Therefore, *Salmonella* can contaminate carcasses at the slaughterhouse, especially if facilities and hygiene are poor: for example, if floors are dirty, or if the same tank filled with dirty water is used for all washing steps. In addition, transport and sale under humid tropical conditions may further facilitate *Salmonella* contamination and growth [16–19]. A recent nationwide survey in Cambodian markets reported that 42.6% of chicken meat samples were contaminated with *Salmonella*, with an average most probable number (MPN) of 10.6 MPN/g [20]; previous studies on retailed chicken meat samples also showed high *Salmonella* prevalences which ranged from 20–60% [21–24].

Chicken is widely used in Cambodian cuisine, including traditional salad, consisting of boiled chicken mixed with raw vegetables, herbs, spices and banana flowers [25]. Chicken salad is commonly prepared in households and restaurants and for wedding banquets. Salad is prone to contamination because it includes raw ingredients, is served cold, and its preparation requires equipment and several handling steps [16]. A common cause of bacterial cross-contamination of ready-to-eat (RTE) foods is poor hygiene practices allowing pathogens from raw meat to contaminate hands, knives and cutting boards and hence be transferred to other food [17, 25, 26]. Unfortunately, food hygiene often receives insufficient attention since Cambodian consumers are more concerned about chemicals than microbes [27]. Yet, considering the high *Salmonella* prevalence in chicken meat, consumers could be at risk from chicken salad. To quantify the risk of foodborne illness from eating chicken salad, information on microbial load and cross-contamination in this typical dish is needed. However, the levels and mechanisms of contamination of Cambodian salad with *Salmonella* have not been investigated. Therefore, this study aimed to investigate how *Salmonella* from raw chicken could cross-contaminate RTE chicken salad, given the usual practices in Cambodian households. These experimental results can model exposure assessment steps in quantitative microbial risk assessment of *Salmonella* in chicken salad and recommend reducing cross-contamination while preparing food.

## Materials and methods

### Household survey on hygiene practices when cooking chicken salad

In 2020, 12 focus group discussions (FGD) with consumers were held in four provinces (Siem Reap, Preah Sihanouk, Battambang and Phnom Penh) in Cambodia. In each province, three areas representing different geographical and social contexts, covering rural, peri-urban and urban areas, were purposely selected based on information from local authorities. In each area, individuals who were mainly in charge of purchasing and preparing food for their families, from six to eight households, were invited by the communal authorities to participate in the FGD. A total of 93 participants discussed food safety practices during purchasing, storing and washing of chicken carcasses and vegetables, use and cleaning of kitchen utensils, and hand washing when preparing the traditional chicken salad *(‘ngam sach man sroyong chek’*). After gaining written consent from the participants, the FGD was facilitated by a senior researcher while another researcher took notes and recorded the discussion. The FGD was conducted in the Khmer language and lasted approximately 1.5 to 2 hours. Transcripts were then translated into English for quantitative analysis.

### Cooking chicken salad scenarios

Cambodian chicken salad is a mix of boiled chicken meat with sliced banana flowers, cucumber, tomato, lemon juice, basil, fresh chili, herbs, and spices (hereafter, these plant ingredients are referred to as ‘vegetables’). Fresh vegetables are washed and cut into small pieces. A whole chicken carcass bought from the market is washed, cut into pieces, boiled, deboned, then torn and sliced into shreds. Boiling lasts around 20 minutes, and the time for total preparation is one to two hours.

**Fig 1:**
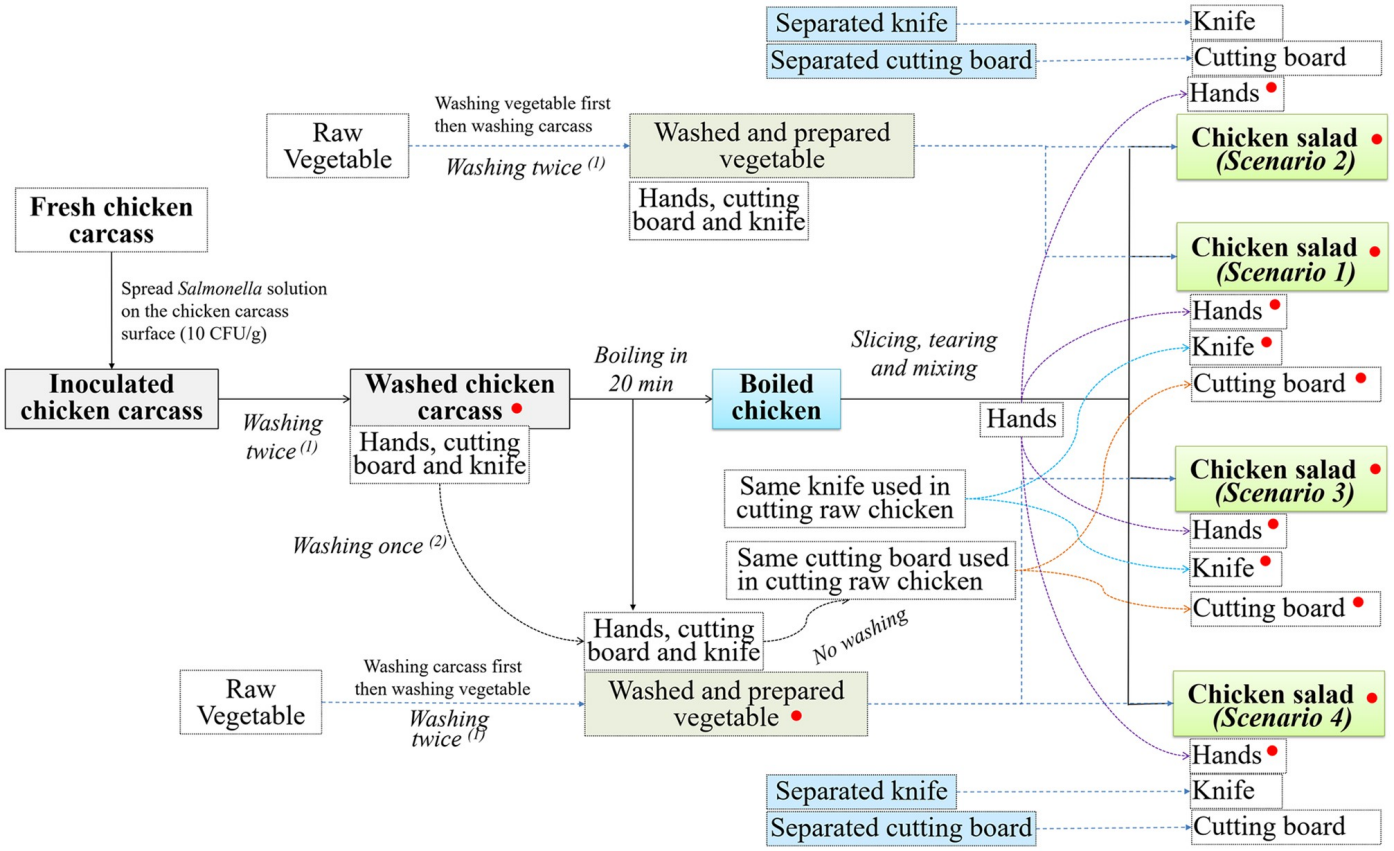
The scenario diagram of the preparation and practice steps of cooking chicken salad, including the sampling points. Red dots indicate the sampling types and stages collected during the experiment of each scenario: ^1^washing with clean, *Salmonella*-free water; ^2^washing with clean, *Salmonella*-free water, dishwashing detergent and clean dishcloth.

Four experimental scenarios were designed to imitate the process of preparing the chicken salad in the household as reported in FGDs (that is, washing vegetables either before or after washing the chicken carcass and using the same knife and cutting board (utensils) for chicken and vegetables or different utensils). All four experimental scenarios were carried out on the same day and repeated nine times per scenario. The main steps of cooking chicken salad in each scenario are described in Table 1 and Fig 1.

**Table 1:** Chicken salad preparation steps in each experimental scenario and the number of samples collected and analyzed.

Practices	Preparation steps in each scenario and number of samples collected (n)				Total samples	*Salmonella* analysis	
	Scenario 1	Scenario 2	Scenario 3	Scenario 4		Qualitative	Quantitative
						(Yes/No)	(MPN/g)
1. Wash vegetables twice with water* and slice into small pieces for salad	●	●					
2. Wash chicken carcass twice with water*	●	●	●	●			
3. Cut chicken carcass into smaller parts^1^	● (9)	● (9)	● (9)	● (9)	36	●	
4. Wash vegetables twice with water and slice into small pieces^2^ for salad			● (9)	● (9)	18	●	
5. Wash used cutting board, knife and hands once with dish detergent and water	●	●	●	●			
6. Boil chicken carcass (20 min) and take out and wait to cool down (40–45 min)	●	●	●	●			
7. Debone and cut the boiled chicken into small pieces and mix with prepared vegetables using the same, but washed, cutting board, knife and hands^3^.	● (27)		● (27)		54	●	
8. Debone and cut the boiled chicken into small pieces and mix with prepared vegetables using a different** cutting board and knife and also washing hands^4^		● (9)		● (9)	18	●	
9. Mix and place ready-to-eat chicken salad on the dish^5^	● (9)	● (9)	● (9)	● (9)	36	●	●
Total samples	45	27	54	36	162		

Note:

* Water for all steps was clean and *Salmonella*-free;

** The cutting board and knife were disinfected to be *Salmonella*-free prior to use in each experiment;

^1^ swab of 25 cm^2^ of chicken surface;

^2^ approximately 50 g of mixed-prepared vegetable was collected;

^3^ a set of surface swab samples including cutting board (25 cm^2^ in the centre), knife (both sides of the blade, 25 cm^2^ each) and hands (palms, fingers and interdigital folds of two hands) was collected right before slicing boiled chicken;

^4^ only swabs of hands (palms, fingers and interdigital folds of two hands) were sampled;

^5^ approx. 50 g of ready-to-eat salad comprising both chicken meat and vegetable was sampled.

#### Scenario 1

*Wash vegetables first and use the same utensils (WVF-SU)*. The process was washing (twice) and chopping vegetables; washing and cutting the raw chicken carcass; washing the cutting board, knife and hands (once with dish detergent); boiling the chicken and using the same (washed) knife, cutting board and hands to debone, tear and slice the boiled chicken and, finally, mixing the salad.

#### Scenario 2

*Wash vegetables first and use different utensils (WVF-DU)*. The process was washing (twice) and cutting vegetables; washing and cutting raw chicken carcass; washing the cutting board, knife and hands (once with dish detergent); boiling the chicken; using a separate knife and cutting board, and washing hands to debone, tear and slice boiled chicken and mixing the salad.

#### Scenario 3

*Wash chicken first and use the same utensils (WCF-SU)*. The process was washing (twice) and cutting raw chicken carcass; washing and chopping vegetables; washing the cutting board, knife and hands (once with dish detergent); boiling the chicken; using the same (washed) knife, cutting board and hands to debone, tear and slice boiled chicken, and mixing the salad.

#### Scenario 4

*Wash chicken first and use different utensils (WCF-DU)*. The process was washing (twice) and cutting the raw chicken carcass; washing and chopping vegetables; washing the cutting board, knife and hands (once with dish detergent); boiling the chicken; using a separate knife and cutting board, and washing hands to debone, tear and slice boiled chicken, and mixing the salad.

### Chicken carcass preparation and inoculation

#### Preparation of the chicken carcass

Four whole chicken carcasses (approximately 1.2 ± 0.2 kg each) were purchased from a shop that had tested negative for *Salmonella* in a recent Cambodian market survey [20]. The butcher was supervised to ensure hygienic processing, including thorough washing and disinfection of hands and equipment (knives and buckets). After cleaning, each chicken carcass was washed thoroughly twice with clean and *Salmonella*-free bottled drinking water (Vital, Phnom Penh, Cambodia) to minimize bacterial contamination. Immediately after that, the washed carcasses were placed into separate sampling bags. The packed carcasses were placed in a cool box and transported immediately to the laboratory to start the experiment within 1 hour.

#### Preparation of *Salmonella* inoculum

According to recent studies in Cambodia and Vietnam, *Salmonella* Typhimurium and *Salmonella* London were commonly found in retailed ASF, including chicken meat and pork [24, 28]. This study utilized two *Salmonella* strains (*S*. Typhimurium and *S*. London) isolated from Vietnam [16] to prepare an inoculation culture. Both strains were cultured separately in a 100 mL glass bottle (Schott Duran, Mainz, Germany) consisting of 50 mL Buffered Peptone Water (BPW; Merck, Darmstadt, Germany) for 12–14 h at 37°C with gentle agitation. Next, the *Salmonella* load in each strain culture was enumerated using a spread plate count method on Xylose-Lysine Deoxycholate Agar (XLD; Hi-Media, Mumbai, India). Based on the determined concentration in each initial cultured strain, a final suspension of 10^4^ *Salmonella* CFU/mL medium was made of appropriate mixed-volume proportions of the two strains. The final *Salmonella* suspension was then used to inoculate the chicken carcass.

#### Inoculation of chicken carcass

Immediately after preparing the *Salmonella* medium (concentration of 10^4^ CFU/mL), each chicken carcass was weighed (in grams) and inoculated with a corresponding volume (in microlitres) of *Salmonella* medium; for instance, a 1,200-gram carcass was inoculated with 1,200 μL *Salmonella* medium. *Salmonella* medium was dropped over the entire chicken carcass surface using 10–200 μL tips and pipette (Corning, NY, US), which created a 10 CFU *Salmonella* per one gram of chicken. After the inoculation, carcasses were kept at room temperature for 30 min for stable absorption, following the methodology of previous experiments [16, 29].

#### Washing of vegetables, chicken carcasses, hands and equipment, and preparation of vegetables and chicken carcasses

Vegetables comprised banana flowers, lemon, fresh chilli, cucumber, tomato and basil bought from a shop in the early morning. Before using, the researchers washed them twice with clean water containing 1% sodium chloride and immersed them in saline water for 30 min to minimize contamination and then cut them into small slices using a washed cutting board, knife and hands.

The inoculated chicken carcass was washed twice in a basin using clean, *Salmonella*-free water (approximately 5 L per chicken) with clean, bare hands. The washed carcass was put on the cutting board and cut into parts (neck-head, two wings, two drumsticks, two thighs and two breasts). The chicken parts were then boiled in a pot with 2–2.5 L of water for 20 min, after which a spoon was used to remove and place them on a sterile plate to cool down for approximately 40–45 min. The boiled chicken was then deboned, sliced and torn into small pieces using clean bare hands, knives and a cutting board. The cutting board, knife and hands used to cut the fresh carcass were washed once in a different basin with clean water using dishwashing detergent (Sunlight, Unilever, Vietnam) and dishcloth (Sunlight, Unilever, Vietnam) for about 3–4 min as described in an earlier study [16]. The cutting board and knife were kept at ambient temperature to dry at least 15 min before the next step. Vegetables were prepared and processed according to four pre-defined scenarios (Table 1).

#### Sampling

In each scenario, just before chopping for boiling, the chicken carcass was sampled using a 5 x 5 cm stainless steel frame and a pre-moistened gauze to swab the breast surface. In scenarios 3 and 4, after washing and cutting, but before mixing with meat, vegetables were sampled by taking approximately 50 g of the mixed vegetables with sterile forceps. In scenario 1 (WVF-SU) and 3 (WCF-SU), just before slicing the boiled chicken, a set of three surface swabs and pre-moistened gauze samples were collected from the cutting board (25 cm^2^ in the centre), knife (both sides of the blade, 25 cm^2^ each) and two hands (palms, fingers, interdigital folds). In scenarios 2 (WCF-SU) and 4 (WCF-DU), the surface of two hands (palms, fingers and interdigital folds) was swabbed just before slicing boiled chicken. After finishing the last step (mixing salad) in each scenario, samples of RTE chicken salad were collected by taking approximately 50 g each of chicken meat and vegetables. The number of samples taken in each step by scenarios and *Salmonella* analyses is presented in Table 1.

#### Microbiological test

Following the ISO procedure, all samples (n = 162, Table 1) underwent *Salmonella* isolation [30]. A sample of 25 g chicken salad or vegetables or swab was homogenized with appropriate BPW volume and incubated for 18±2 h at 37°C as a pre-enrichment step. Selective enrichment step was done by adding 1 mL of suspension into 9 mL Muller Kauffmann Tetrathionate (MKTT; Merck, Germany) incubated for 24 ± 3 h at 37°C and 0.1 mL into 10 mL Rappaport-Vassiliadis Soya (RVS; Merck, Germany) incubated for 24 ± 3 h at 41.5°C. Selective plating was performed by streaking one loopful (approximately 10 μL) each of MKTT and RVS onto XLD agar. MacConkey (Merck, Darmstadt, Germany) was the second selective plating agar. Two presumptive *Salmonella* colonies per plate were selected to test biochemically (lactose, indole, lysine and hydrogen sulfide) for *Salmonella* confirmation.

A 3-tube MPN method was used to quantify *Salmonella*, as described previously [31]. In brief, the sample was diluted first at 10^−1^ by adding 25 g of chicken salad in 225 mL BPW. Three tubes containing 10 mL of this 10^−1^ dilution were prepared, after which 1 mL of the 10^−1^ dilution was added to three tubes containing 9 mL BPW to make the second series of 10^−2^; then 1 mL of 10^−2^ was added to the last three tubes containing 9 mL of BPW to make the third series of 10^−3^. The three-tube set of three consecutive dilutions was incubated at 37°C for 18 ± 2 h. The steps to detect *Salmonella* in each tube were followed according to the isolation procedures mentioned above. The presence of *Salmonella* in the three tube-set was used to calculate the MPN index according to the method described earlier [32, 33].

#### Data analysis and modelling

Data were entered into Excel spreadsheets (Microsoft, 2016) and analyzed descriptively (proportion, mean, standard deviation). A chi-squared test or Fisher exact test was used to evaluate the *Salmonella* cross-contamination proportions among sample types and scenarios. R version 3.3.2 (R Core Team, 2020) was used to compute testing and bootstrapping [34]. To describe the distributions of *Salmonella* concentration in the chicken salad in each scenario, both non-parametric and parametric bootstrapping techniques were used. *Salmonella* was quantified as MPN/g and thus followed log-normal distribution. A Bayesian statistic was used to assess variability and uncertainty during the simulation of *Salmonella* load and reduction rates. The parameters and functions used to carry out the bootstrapping, and simulated sample data distributions followed the steps described earlier [16]. The function fitdist() in the *fitdistrplu*s package in R was used to estimate the mean and standard deviation of *Salmonella* CFU/g [34]. For the presentation of distributions, kernel density was calculated in density() function based on the simulated sample data and plotted using R. We model the reduction of *Salmonella* CFU/g by using the equation: *Reduction rate = (10—Salmonella CFU/g in RTE chicken salad)/10 x 100*, where 10 was an inoculated CFU/g in raw chicken carcass from the beginning of the test. The distribution of the reduction rate was calculated using iterations and presented in a histogram. The experiments showed four MPN values (110, 110, 15 and 15 MPN/g in scenario 1) which were higher than the inoculation level. The simulation performed without and with these four values was named Scenario 1 and the worst-case Scenario 1, respectively. The simulation was not carried out for Scenario 4 since no *Salmonella* positive salad samples were found in this scenario.

#### Ethical statement

The experiments were conducted at the National Animal Health and Production Research Institute (Phnom Penh, Cambodia). Participants invited to the focus group discussions were asked for consent before starting (S1 File). All information on the participants was used among the research team only and not shared with any third party. Written consent was obtained from researchers participating in the experiment, including instructions on safety procedures (S2 File). The chicken salad was sterilized and hygienically discarded after finishing the experiment. Ethical approvals of this study were under the Safe Food, Fair Food Cambodia project and granted by the National Ethical Committee of Cambodia (S3 File), No. 300NECHR, dated 26^th^ December 2017, and the International Livestock Research Institute Institutional Research Ethics Committee (S4 File), No. ILRI-RC010 18/IBC/010/CR, dated 5^th^ July 2018.

## Results

### Hygiene practices when preparing and cooking chicken salad in Cambodian households

Most (86%, 80/93) households reported that they first washed chicken carcasses two to three times with water before washing and preparing vegetables; only 14% (13/93) washed and prepared vegetables before washing chicken carcasses. All participants washed knives and cutting boards at least once, with soap or dishwashing detergent, immediately after cutting fresh chicken carcasses. However, almost all (97%, 90/93) used the same knife and cutting board to prepare raw vegetables and chicken carcasses, as well as to prepare raw and boiled chicken, while the use of separate knives and cutting boards between raw and cooked chicken was less common (3.2%, 3/93, Table 2).

**Table 2:** Food safety practices for preparing chicken salad in Cambodian households.

Practice steps	No. of households (n = 93)	Steps in experiment scenarios
**Store or process after buying raw chicken from the market** (Yes*, %)		
Start cooking immediately after getting home	53 (57.0)	Keep at room temperature for 30 to 45 mins during preparation in all scenarios
Keep at room temperature	31 (33.3)	
Keep in the refrigerator	9 (9.7)	
**The sequence of washing vegetables/herbs and raw chicken** (Yes, %)		
Wash vegetables first, then wash the chicken carcass	13 (14.0)	Applied in scenarios 1, 2
Wash chicken carcass first, then wash vegetables	80 (86.0)	Applied in scenarios 3, 4
**Number of times to wash chicken carcass before processing** *(*time, *mean (min-max))*	2.9 (1–5)	Applied to wash carcass two times in all scenarios
**Wash knives, cutting board and hands with soap/dish detergent after cutting chicken carcass** *(Yes*, *%)*	93 (100)	Applied to wash hands, equipment in all scenarios
**The average number of times when washing knives, cutting board, hands after cutting raw chicken** *(times*, *mean (min-max))*	1.3 (1–3)	Applied to wash one time in all scenarios
**Length of boiling chicken for salad dish counted from boiling stage** *(*minutes, *mean (min-max))*	29 (15–60)	Applied to boil chicken in 20 mins in all scenarios
**Use the same knife and cutting board with washing once in between when preparing raw and cooked chicken** *(Yes*, *%)*	90 (96.8)	Applied in scenarios 1, 3
**Use the same knife and cutting board with washing once in between when preparing raw vegetable and chicken carcass** *(Yes*, *%)*	90 (96.8)	Applied in scenarios 3, 4

* Yes versus No *(“No” means using separate knives and cutting boards between vegetable and meat*, *or between raw and cooked meat*, *but hands were washed once with soap or detergent)*.

### *Salmonella* contamination from the raw chicken after washing to vegetables/herbs, hands, cutting board and knife during chicken salad preparation

**Fig 2:**
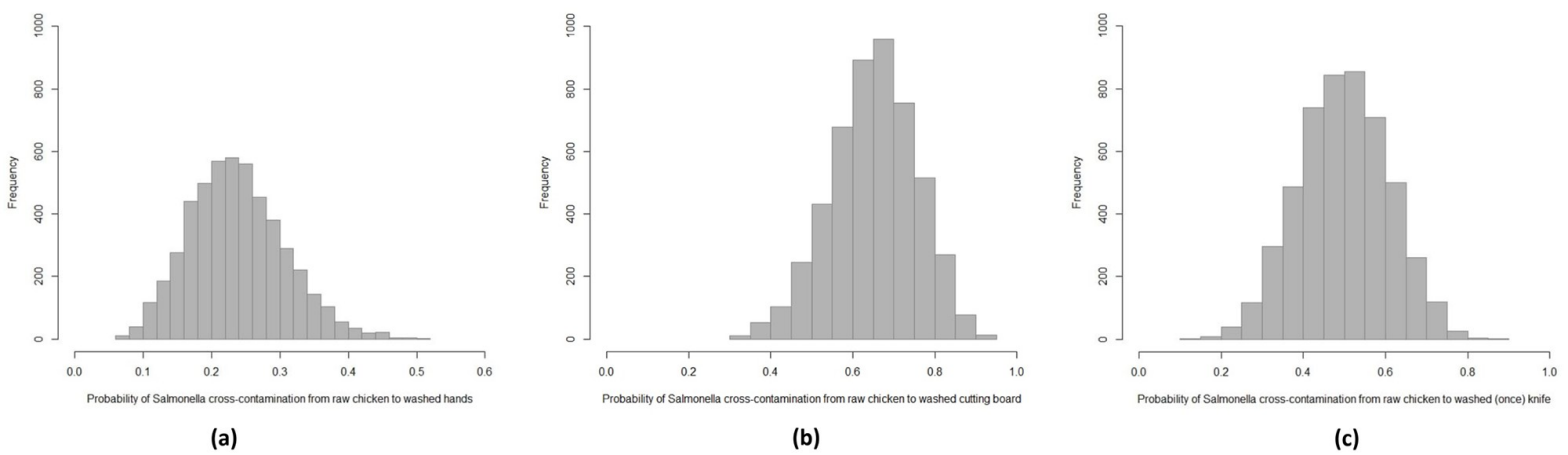
Simulated probability distribution of *Salmonella* cross-contamination from raw chicken to hands (a), cutting board (b) and knife (c) after washing once after washing and cutting the fresh chicken carcass.

After washing the chicken carcasses twice, *Salmonella* was isolated from 32 out of 36 samples (88.9%, 95%CI: 73.0–96.4). Two out of 18 vegetable samples were cross-contaminated with *Salmonella* (11.1%, 95%CI: 1.9–36.1, Table 3). Eight out of 36 hand swabs (palms, fingers and interdigital folds of two hands) (22.2%, 95%CI: 10.7–39.6), which had been washed once after handling contaminated chicken carcasses, were positive for *Salmonella*, which was significantly lower compared to 66.7% of washed cutting boards being contaminated (12/18, χ2 = 8.35, df = 1, p-value = 0.004) and lower compared to contamination on washed knives (50.0%, 9/18, χ2 = 3.10, df = 1, p-value = 0.07, Table 3). *Salmonella* from chicken carcasses was most often transferred to the cutting boards, followed by knives and hands, even though hands and equipment were washed once with clean water and dishwashing detergent. From the simulated data, *Salmonella* cross-contamination to cutting boards was significantly higher than to knives and hands and was higher to knives than to hands (*p*-value < 0.001); average contamination on hands, knives and cutting boards was 23.7% (95%CI: 5.1–28.3), 50.1% (95%CI: 42.5–57.8) and 65.2% (95%CI: 58.3–72.6), respectively (Table 3, Fig 2).

**Table 3:** *Salmonella* contamination from the raw chicken after washing twice and vegetables, hands, knives and cutting boards during chicken salad preparation.

Sample types	Experimental data		Simulated data*	
	No. of *Salmonella* positive (Scenario 1/2/3/4)*/*No. of experiments	Contamination percentage (%, 95%CI)	No. of *Salmonella* positive*/*No. of iterations	Contamination percentage (%, 95%CI)
*Salmonella* contamination in raw chicken and vegetables** **after washing twice**				
Washed raw chicken carcasses	32 (8/7/8/9)/36	88.9 (73.0–96.4)	4340/5000	86.8 (83.5–90.9)
Washed and prepared vegetables	2 (na/na/1/1)/18	11.1 (1.9–36.1)	745/5000	14.9 (9.1–19.6)
**Cross-contamination of *Salmonella* to hands, cutting boards and knives** ***				
Washed hands after handling contaminated chicken carcasses	8 (3/1/3/1)/36	22.2 (10.7–39.6)^a^	1185/5000	23.7 (5.1–28.3)^a^
Washed knives after handling contaminated chicken carcasses	9 (3/na/6/na)/18	50.0 (29.0–70.9)^a^	2505/5000	50.1 (42.5–57.8)^b^
Washed cutting boards after handling contaminated chicken carcasses	12 (5/na/7/na)/18	66.7 (41.2–85.6)^b^	3260/5000	65.2 (58.3–72.6)^c^

Note: “na”: Not applicable; Different superscript letters in the same column indicate significant difference; CI: confidence interval;

* Simulated data were generated from random sampling 5000 times, in which initial values were based on experiment samples and positive numbers using beta distribution in RStudio: [rbeta(5000, positive+1, n-positive+1)];

** Vegetables (included banana flower, lemon, fresh chilli, cucumber, tomato and basil) were washed twice with clean Salmonella-free water using the same knives, cutting boards and hands for preparation (i.e. cutting);

*** Knives, cutting boards and hands were washed once using clean Salmonella-free water and dishwashing detergent after cutting the raw chicken carcasses.

### Cross-contamination of *Salmonella* to ready-to-eat chicken salad

In Scenario 1 (WVF-SU), seven out of nine (77.8%) chicken salads were positive with *Salmonella*, while the number of salad samples positive with *Salmonella* in Scenarios 2 (WFV-DU) and 3 (WCF-SU) was one out of nine (11.1%) and two out of nine (22.2%), respectively. Scenario 4 (WCF-DU) showed no positive *Salmonella* in salad in all nine experiments. Scenarios 1 and 3 used the same knife, cutting board, and hands for handling salad, while in Scenarios 2 and 4, a separate knife and cutting board were used. There was a significantly higher cross-contamination rate in Scenario 1 compared to the other three scenarios (Fisher exact test, *p*-value = 0.05). The average *Salmonella* contamination in the salad was highest in Scenario 1 (37.3 MPN/g) and 0.36 MPN/g in both Scenarios 2 and 3. The overall proportion of *Salmonella* contamination in all four scenarios was 27.8% (95%CI: 14.8–45.4, Table 4).

**Table 4:** Contamination of *Salmonella* in ready-to-eat chicken salad during preparation and handling in four different experiment scenarios.

Scenario	No. of *Salmonella* positive/Total samples	Proportion of contamination	95%CI	*Salmonella* MPN/g (mean, min-max)
Scenario 1 (WVF-SU)	7/9	77.8^a^	40.2–96.1	37.3 (0.1–110)
Scenario 2 (WVF-DU)	1/9	11.1^b^	0.6–49.3	0.36 (0.36–0.36)
Scenario 3 (WCF-SU)	2/9	22.2^b^	3.9–39.8	0.36 (0.36–0.36)
Scenario 4 (WCF-DU)	0/9	0.0^b^	0.0–37.1	NA
**Overall**	**10/36**	**27.8**	**14.8–45.4**	**26.2 (0.1–110)**

Note: Different superscript letters in the same column indicate significant differences; CI: confidence interval; NA: not available.

**Fig 3:**
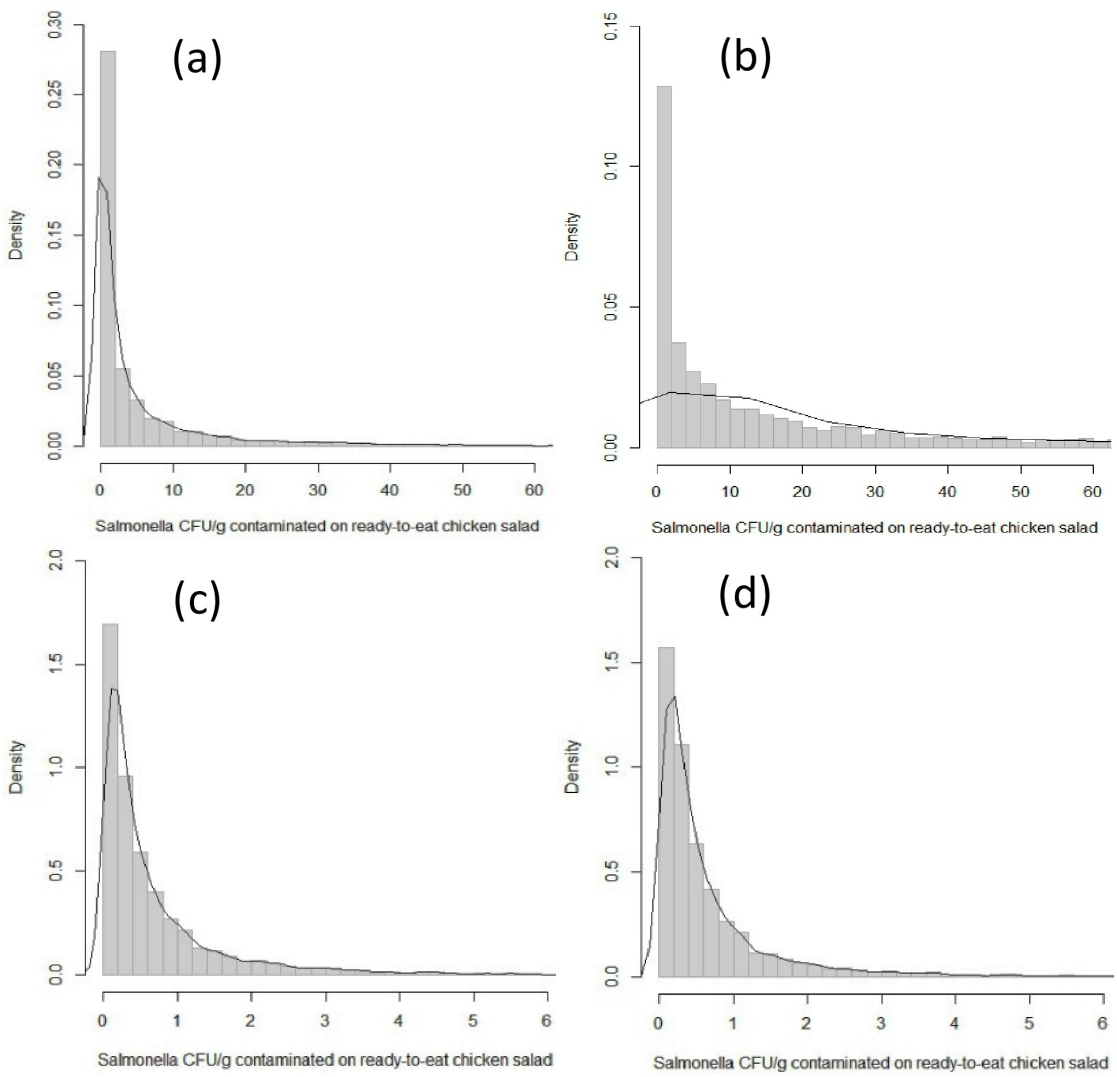
*Salmonella* concentration (CFU/g) on contaminated RTE chicken salad in Scenario 1* (a), Scenario 1** (b), Scenario 2 (c) and Scenario 3 (d) based on the experiment values simulated 5000 times. * Scenario 1 had only values below the initial concentration (10 CFU/g); ** worst-case Scenario 1 included four MPN/g values (two 110 CFU/g and two 15 CFU/g), which exceeded the initial concentration (10 CFU/g).

Based on simulated data (5000 iterations), the average *Salmonella* contamination in salad was higher in Scenario 1 (8.58 CFU/g) (including 4 values that exceeded the initial value of 10 CFU/g) with a mean of 77.78 CFU/g. In contrast, the average *Salmonella* contamination in Scenarios 2 and 3 was 0.8 and 0.78 CFU/g, respectively (Table 5 and Fig 3). (*Salmonella* was absent in Scenario 4).

**Table 5:** *Salmonella* (CFU/g) concentration was simulated in each scenario in the ready-to-eat chicken salad, based on the experiment values with 5000 iterations.

Scenario	The concentration of *Salmonella* (CFU/g)* contaminated with chicken salad			
	Mean	Median	Lower limit	Upper limit
Scenario 1 (WVF-SU)**	8.58	1.31	0.002	59.15
Scenario 1***	77.78	12.41	0.008	577.0
Scenario 2	0.80	0.36	0.032	4.09
Scenario 3	0.78	0.36	0.029	4.03

* CFU: Colony-forming unit;

** Scenario 1 had only values below the initial concentration (10 CFU/g);

***Worse-case Scenario 1 included four MPN/g values (two 110 CFU/g and two 15 CFU/g) which exceeded the initial concentration (10 CFU/g).

### The reduction rate of *Salmonella* contamination from raw chicken to ready-to-eat chicken salad

**Fig 4:**
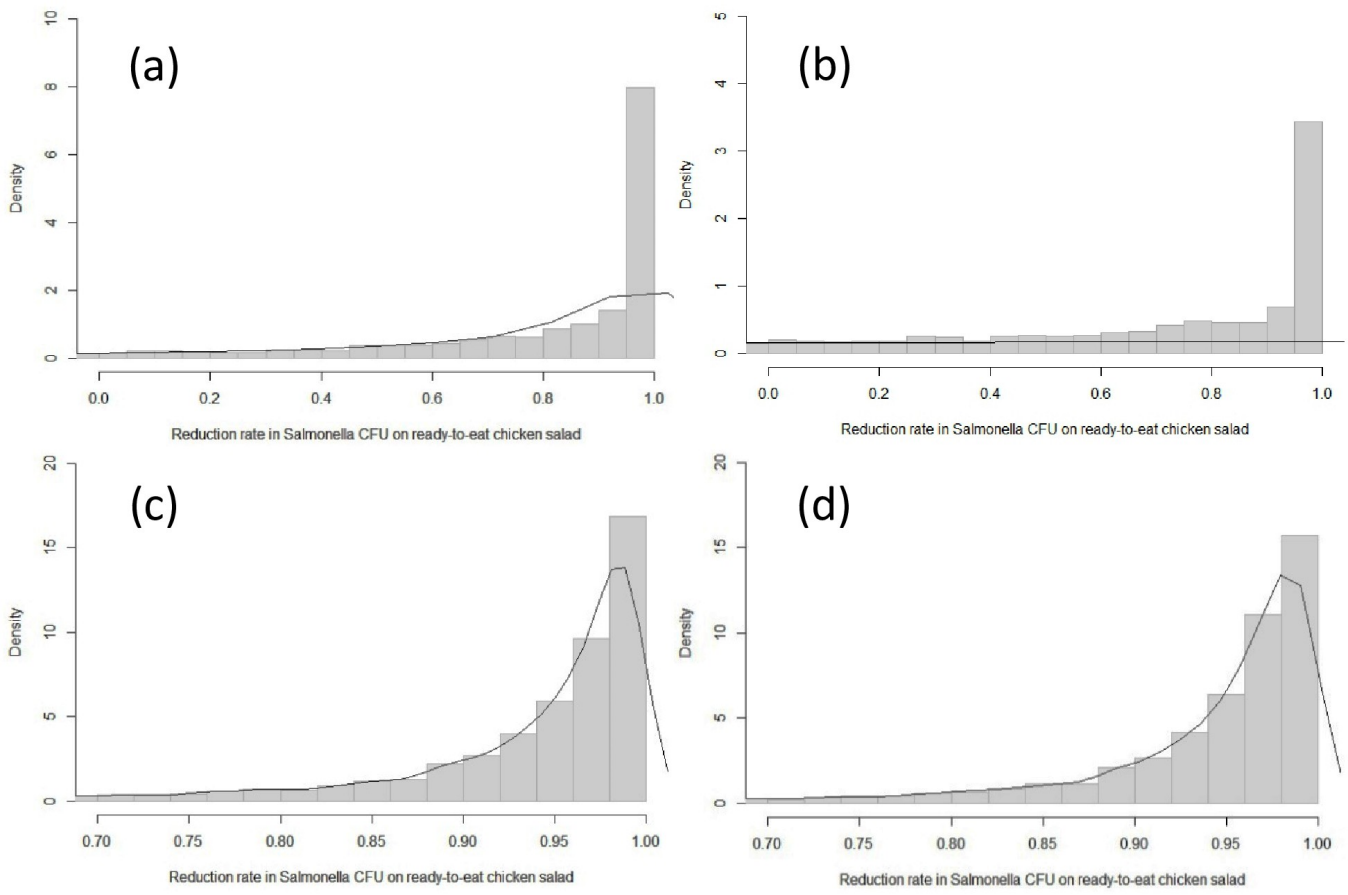
The reduction rate of *Salmonella* concentration (CFU/g) in the contaminated RTE chicken salad in Scenario 1* (a), worst-case Scenario 1** (b), Scenario 2 (c) and Scenario 3 (d) based on the experiment values to bootstrap 5000 times. * Scenario 1 had only values below the initial concentration (10 CFU/g); ** Worst-case Scenario 1 included four MPN/g values (two 110 CFU/g and two 15 CFU/g), which exceeded the initial concentration (10 CFU/g).

Most *Salmonella* was transmitted from raw chicken to salad in Scenario 1 (WVF-SU). In this scenario, four out of seven positive samples had higher levels of *Salmonella* after processing. On the other hand, in Scenarios 2 (WVF-DU) and 3 (WCF-SU) *Salmonella* was reduced by 92%. The proportion of the simulated values that exceeded initial CFU/g in Scenarios 1, 2 and 3 were 53.8, 18.6, 3.8 and 3.6, respectively (Table 6 and Fig 4).

**Table 6:** Simulated reduction rate (percentage) of *Salmonella* concentration (CFU/g) in the RTE chicken salad in each scenario based on the experiment values simulated 5000 times.

Scenario	The reduction rate of *Salmonella* concentration, CFU/g*				
	Mean	Median	Lower limit	Upper limit	Exceeded initial CFU/g
Scenario 1	14.2	86.9	-504	99.9	18.6
Worst-case Scenario 1**	-728.2	-24.1	-6241.1	99.9	53.8
Scenario 2	92.0	96.4	58.5	99.7	3.8
Scenario 3	92.2	96.4	59.7	99.7	3.6

* CFU: Colony-forming unit;

**Worse-case Scenario 1 included four MPN/g values (two 110 CFU/g and two 15 CFU/g) which exceeded the initial inoculated concentration (10 CFU/g).

## Discussion

This study examined how food handling might affect the risk of cross-contamination by *Salmonella* in households under different preparation scenarios. The results indicate interventions to reduce risk. In addition, findings can be used to model exposure assessment steps in conducting a quantitative microbial risk assessment of *Salmonella* in chicken salad. In Cambodia, several foodborne outbreaks have been associated with contamination during food preparation [5, 35]. The FGDs of the 93 households described the common practices that were used to develop scenarios for the experiment. Chicken and pork salad are typically consumed in households, restaurants or ceremonies in Cambodia [36–38], Southeast Asia and Middle Eastern countries [39]. FBD cases have been associated with these [35, 36]. *Salmonella* in raw chicken carcasses sold in the market is a source of contamination, especially when using the same hands, knife, or cutting board without adequate washing [40]. Several factors could contribute to the current high prevalence of *Salmonella* in retailed chicken and pork in Cambodian markets (exceeding 40%) [20]. These include reused or unsafely used water for cleaning (for example, washing intestines in the same basin as carcasses), absence of appropriate storage facilities for food, low frequency of cleaning and disinfection of the shop, etc. [6, 14, 41]. Despite washing the chicken carcasses twice in water, this study found that 88.9% (95% CI: 73.0–96.4) of experimental carcasses still harboured *Salmonella*, similar to a finding in an experiment of washing contaminated pork in Vietnam, but the washing steps significantly reduced the number of *Salmonella* bacteria by up to 92.2% (Table 5) [16, 42, 43]. In this study, handling and preparing raw meat (including washing and cutting) increased *Salmonella* contamination of hands, cutting boards, knives and vegetables. Washing of food products before preparation is often observed in many low- and middle -income countries [39] and was also a common practice in the households interviewed in the present study.

In high-income countries, washing raw meat before cooking is usually not recommended because the meat is often contaminated with bacteria, and washing raw meat can spread pathogens around the kitchen. On the other hand, in developing countries, it is often believed that washing meat before cooking removes dirt, slime and blood and makes it safer. In Cambodia, only a few supermarkets or minimarts in urban areas provide packed and cooled or chilled meat and the level of hazards present in these is not well established. Indeed, studies have found that contamination levels are not always lower in modern retail shops in developing countries [20, 43]. Nevertheless, separating equipment used for raw and RTE foods is strongly recommended in all contexts. Cleaning and disinfection of hands, knives and cutting board surfaces right after contact with raw meat/chicken can minimize contamination with bacteria [43].

The current study detected *Salmonella* cross-contamination from chicken meat to RTE Cambodian chicken salad, with 27.8% of all salads being contaminated. This is the first study to quantify cross-contamination of bacteria during simulated home preparation of ASF products in Cambodia. Studies in other countries on cross-contamination from pork [16] and chicken [29] found a similar trend of *Salmonella* cross-contamination to RTE food. *Salmonella* from ASF can contaminate hands, equipment and containers and cross-contaminate RTE chicken salad during preparation, consequently causing foodborne illness in consumers. Several reports show that FBDs, especially in developing countries, are often underreported, and there is a lack of food safety surveillance and traceability systems [1, 3, 5–7, 44]. This implies there are more foodborne illness cases, including salmonellosis, in Cambodia than the number officially reported by the health authority (5000 cases of FBD in 2019) [5, 35].

Scenarios 1 (WVF-SU) and 3 (WCF-SU), using the same utensils for chopping chicken and vegetables and assumed to be the scenarios with the poorest hygiene practices, had the highest proportion of *Salmonella* contamination of salad (77.8% and 22.2%, respectively) and the highest quantity of *Salmonella* (37.3 MPN/g, Scenario 1). About 90% of the surveyed households practiced these sub-optimal procedures for preparing chicken salad in their homes. Other studies have also found that unwashed knives, cutting boards and hands increased the risk of cross-contamination in frequency and CFU/g [16, 41, 45]. For example, a Cambodian study of a foodborne outbreak revealed that the unhygienic practices could have led to cross-contaminated food [36]. Cross-contamination of bacteria to RTE food was also reported in studies in China, Indonesia, Malaysia, Thailand and Vietnam [17, 46], where daily food preparation practices were examined. In a similar experiment comparing *Salmonella* contamination when using the same or different utensils to prepare boiled pork in Vietnam, the *Salmonella* prevalence varied from 22.2–77.8%, and average *Salmonella* concentrations were from 0.12 to 5.79 CFU/g in cooked pork [16].

This study shows that washing did not eliminate *Salmonella* from the chicken. In addition, washing vegetables after washing the raw chicken carcasses resulted in the transfer of *Salmonella* to the vegetables via hands or equipment, or both. However, we did not expect that levels of *Salmonella* in the chicken salad in scenario 1 (vegetables washed first) would be worse than in scenario 3 (chicken washed first). In addition, in Scenarios 3 and 4 of our experiment, two out of eighteen washed and prepared vegetables were positive with *Salmonella* (Table 3) would explain the less involvement of hands and sink in transferring *Salmonella* to vegetables during washing contaminated chicken first. Practices of washing chicken carcass before washing vegetables were reported in 86% of interviewed households (Table 2); however, actual of using the sink, basket, hands, or contact with rinsed water in washing vegetables can be different by households thus, the level of cross-contamination to vegetables could be higher than in our experiment. Washing meat could decrease bacterial contamination; nonetheless, the varied practices also result in different levels of bacteria remaining in food [42]. Cutting boards have been reported to contribute the most to the cross-contamination of bacteria from raw ASF to other food [28]. A study in China using *Campylobacter* spp. as an indicator of in-home cooking procedures also found that cutting boards were an important source of cross-contamination [47].

Scenarios 2 (WVF-DU) and 4 (WCF-DU) were more hygienic as a separate knife and cutting board were used to prepare raw and cooked chicken. A lower proportion of *Salmonella*-contaminated chicken salads was observed in scenarios 2 and 4 (11.1% and 0.0%, respectively) than in scenarios 1 (WVF-SU) and 3 (WCF-SU). However, there was only one *Salmonella-*positive vegetable sample in scenario 3 and one in scenario 4 (WCF-DU) when vegetables were washed and chopped after the chicken. It is generally agreed that washing vegetables before handling and preparing raw meat would significantly reduce the risk of cross-contamination [42, 43]. Our study did not find any evidence of this, with scenario 1 having the highest contamination despite washing vegetables first and scenario 4, with chicken, washed first, resulting in no contamination of the chicken salad.

Information on *Salmonella* prevalence, load and reduction rate in each hygiene practice scenario, will be helpful in exposure assessment; a step often inadequately addressed in risk assessment [48]. This is the first study investigating cross-contamination by *Salmonella* in Cambodian households when preparing salad. The findings show different ways that bacteria can contaminate RTE food and may be generalized to other types of salad prepared by similar procedures and used to assess the risk of cross-contamination in other types of raw meat and seafood. Furthermore, the results can be used to design and disseminate more targeted, evidence-based food safety practice messages, such as the need to use a separate cutting board and knife for raw and RTE food and adequately clean and disinfect hands and equipment surfaces after contact with raw meat.

This study had some limitations. The experiment used raw chicken and ‘vegetables’ (cucumber, tomato, basil, fresh chili and banana flower) purchased from hygienic slaughterhouses and shops; however, these might not always have been *Salmonella*-free during all nine experimental days. In addition, during the experiment, the variation in contact time, pressure and moisture and the nature (surface) of meat, vegetables and equipment between replications might have affected *Salmonella* (cross) contamination [49–51]. Future studies should also assess the risk of washing instead of not washing chicken carcasses before cooking.

## Conclusions

Our finding that the median *Salmonella* load in the chicken salad was between 0.36 and 12.41 CFU/g raises health concerns. We described the usual practices of preparing chicken salad in Cambodia and examined how these could lead to *Salmonella* being transferred from chicken carcasses to salad, identifying risky practices including the use of the same cutting board and knife for meat and vegetables and inadequate handwashing. Different salad preparation practices result in very different contamination levels, with washing vegetables before the chicken and using the same utensils for chopping chicken and vegetables resulting in higher levels of salad contamination. Risk communication messages should focus on the need for separate kitchen utensils and frequent and adequate washing and disinfecting of food contact surfaces (cutting board, knife, hands). The finding that washing chicken carcass before the vegetables resulted in less contamination was not expected and requires further investigation. Data on *Salmonella* levels under different preparation scenarios will be used to support quantitative microbial risk assessments through eating salad.

## Supporting information

S1 File Consent form for participant in research: Household. (PDF)

S2 File Consent form for participant in research: Laboratory research team. (PDF)

S3 File Ethical approvals of this study granted by the National Ethical Committee of Cambodia. (PDF)

S4 File Biosafety check by International Livestock Research Institute Institutional Research Ethics Committee. (PDF)

S5 File Funding statement. (PDF)
