# Supplementary material for: Experimental cross-contamination of chicken salad with Salmonella enterica serovars Typhimurium and London during food preparation in Cambodian households
Source: PLoS One. 2022 Aug 1;17(8):e0270425. doi: 10.1371/journal.pone.0270425 (PMC9342772; doi:10.1371/journal.pone.0270425)
Supplement: S1 File — (PDF) [file pone.0270425.s001.pdf]

National Ethics Committee for Health Research (NECHR) in Cambodia

Consent form of “Safe Food Fair Food for Cambodia” research project

By Delia Grace, Tum Sothyra, Chhay Ty et al.

CONSENT FORM FOR PARTICIPATION IN RESEARCH

Research Component: Household Food Consumption Behaviour

by interview

I ...., being over 18 years old, hereby consent to participate in the research “Safe food fair food for Cambodia”. I have been given information about the objectives of the research. The details of procedures and any risks have been explained to my satisfaction.

I agree to participate in the research and give my information related to the research objectives on my family's socio-economic characteristics, including housing and assets, and my practice in food handling, consumption, storage, preparation and consumption. I understand that I may not directly benefit from taking part in this research. I am free to withdraw from the project at any time and this will not affect me now or in the future.

I have been informed that, while the information gained in this study will be published, I will not be identified, and individual information will not be divulged.

Name : ………………………….

Signature : ………………………….

Date : ………………………….
