# Supplementary material for: Experimental cross-contamination of chicken salad with Salmonella enterica serovars Typhimurium and London during food preparation in Cambodian households
Source: PLoS One. 2022 Aug 1;17(8):e0270425. doi: 10.1371/journal.pone.0270425 (PMC9342772; doi:10.1371/journal.pone.0270425)
Supplement: S2 File — (PDF) [file pone.0270425.s002.pdf]

<u>National Ethics Committee for Health Research (NECHR) in Cambodia</u>

Consent form of "Safe Food Fair Food for Cambodia" research project

By Delia Grace, Tum Sothyra, Chhay Ty et al.

CONSENT FORM FOR PARTICIPATION IN RESEARCH

Research Component: Experiment of Salmonella enterica cross-contamination during handling and preparation of chicken salad in Cambodian households

Laboratory Research Team

I ...., being over the age of 18 years hereby consent to participate in research "Safe food fair food for Cambodia", in the component of cost of illness study. I have been given information about the objectives of the research. The details of procedures and any risks have been explained to my satisfaction.

I agreed to participate in the research and understand the risk of the experiment. I understand that I may not directly benefit from taking part in this research. I am free to withdraw from the project at any time and this will not affect me now or in the future.

I have been informed that, while the information gained in this study will be published, I will not be identified, and individual information will not be divulged.

Name : ………………………….

Signature : ………………………….

Date : ………………………….
