# Supplementary material for: Experimental cross-contamination of chicken salad with Salmonella enterica serovars Typhimurium and London during food preparation in Cambodian households
Source: PLoS One. 2022 Aug 1;17(8):e0270425. doi: 10.1371/journal.pone.0270425 (PMC9342772; doi:10.1371/journal.pone.0270425)
Supplement: S5 File — (PDF) [file pone.0270425.s005.pdf]

Acknowledgements

This study was carried out under the Safe Food, Fair Food for Cambodia project, funded by the American people through the United States Agency for International Development (USAID) and its Feed the Future Innovation Lab for Livestock Systems managed by the University of Florida. It was also supported by the CGIAR Research Program on Agriculture for Nutrition and Health (A4NH), led by the International Food Policy Research Institute (IFPRI).
