# Supplementary material for: Experimental cross-contamination of chicken salad with Salmonella enterica serovars Typhimurium and London during food preparation in Cambodian households
Source: PLoS One. 2022 Aug 1;17(8):e0270425. doi: 10.1371/journal.pone.0270425 (PMC9342772; doi:10.1371/journal.pone.0270425)
Supplement: S3 File — (PDF) [file pone.0270425.s003.pdf]

ILRI
INTERNATIONAL LIVESTOCK RESEARCH INSTITUTE

CGIAR

5th July, 2018

Our Ref: ILRI-RC010 18/IBC/010/CR

International Livestock Research Institute,
P.O. Box 30709-00100,
Nairobi, Kenya.

Dear Dr. Delia Grace

RE: Multi-hazard survey in markets in Cambodia and food safety risk assessment in pork and poultry meat.

This is to inform you that the ILRI IBC has reviewed the risk assessment for the study titled **"Multi-hazard survey in markets in Cambodia and food safety risk assessment in pork and poultry meat"** and granted approval for implementation as per the research protocol and other related documents submitted. The approval period is from 5th July, 2018 to 31st October, 2020.

This approval is subject to compliance with the following requirements:

- Only documents submitted, reviewed and approved by the ILRI IBC shall be used.
- All changes (amendments, deviations, violations) shall be submitted for review and approval by ILRI IBC before implementation.
- Adverse events or unexpected occurrences, related to the study must be reported to the EOHS office immediately.
- All safety requirements as per the risk assessment and protocol submitted shall be adhered to.

- All staff working on the project shall be trained.

Should you need any further clarification, contact ILRI EOHS.

Yours Sincerely,

Dr. Vish Nene

ILRI IBC CHAIR
