# Supplementary material for: Experimental cross-contamination of chicken salad with Salmonella enterica serovars Typhimurium and London during food preparation in Cambodian households
Source: PLoS One. 2022 Aug 1;17(8):e0270425. doi: 10.1371/journal.pone.0270425 (PMC9342772; doi:10.1371/journal.pone.0270425)
Supplement: S4 File — (PDF) [file pone.0270425.s004.pdf]

ព្រះរាជាណាចក្រកម្ពុជា
KINGDOM OF CAMBODIA
ជាតិ សាសនា ព្រះមហាក្សត្រ
NATION RELIGION KING

ក្រសួងសុខាភិបាល
MINISTRY OF HEALTH
គណៈកម្មាធិការជាតិក្រមសីលធម៌
សំរាប់ការស្រាវជ្រាវសុខភាពដែលទាក់ទងនឹងមនុស្ស
National Ethics Committee for Health Research

លេខ......300NECHR..........

រាជធានីភ្នំពេញ, ថ្ងៃទី..26.....ខែ..12.......ឆ្នាំ2017......

Dr. Delia Grace

Project: Safe Food Fair Food For Cambodia. Version N° 1, dated 14th October 2017.

Reference : - 31 October 2017 NECHR meeting minute
- Your clarification letter on 21st December 2017

Dear Dr. Delia Grace

I am pleased to notify you that your clarification of the project entitled "Safe Food Fair Food For Cambodia. Version N° 1, dated 14th October 2017" has been approved by National Ethic Committee for Health Research (NECHR). This approval is valid for twelve months after the approval date.

The Principal Investigator of the project shall submit following document to the committee's secretariat at the National Institute of Public Health at #2 Kim Il Sung Blvd, Khan Tuol Kok, Phnom Penh. (Tel: 855-23-880345, Fax: 855-23-881949):

- Annual progress report
- Final scientific report
- Patient/participant feedback (if any)
- Analyzing serious adverse events report (if applicable)

The Principal Investigator should be aware that there might be site monitoring visits at any time from NECHR team during the project implementation and should provide full cooperation to the team.

Regards,

Chairman

Prof. ENG HUOT

ឡូត៍លេខ៨០.វិថីសម្តេច ប៉ែន នុត (២៨៩) សង្កាត់បឹងកក់២ ខណ្ឌ ទួលគោក រាជធានីភ្នំពេញ. ទូរស័ព្ទ (៨៥៥-២៣) ៨៨០ ៣៤៥. ទូរស័ព្ទដៃ (៨៥៥-១២) ២៨០ ៧៩០. (៨៥៥-១២) ៨៤២ ៤៤២
Lot #80, Samdach Penn Nouth Blvd (289), Sangkat Boeungkok2, Khan Tuol Kork, Phnom Penh, Cambodia. Tel: (855-23) 880 345, Mobile Phone : (855-12) 280 790, (855-12) 842 442
